# Notch Signaling Contributes to Liver Inflammation by Regulation of Interleukin-22-Producing Cells in Hepatitis B Virus Infection

**DOI:** 10.3389/fcimb.2016.00132

**Published:** 2016-10-17

**Authors:** Xin Wei, Jiu-Ping Wang, Chun-Qiu Hao, Xiao-Fei Yang, Lin-Xu Wang, Chang-Xing Huang, Xue-Fan Bai, Jian-Qi Lian, Ye Zhang

**Affiliations:** ^1^Center for Infectious Diseases, Tangdu Hospital, Fourth Military Medical UniversityXi'an, China; ^2^Department of Infectious Diseases, Xijing Hospital, Fourth Military Medical UniversityXi'an, China

**Keywords:** hepatitis B virus, Notch signaling, interleukin-22, innate lymphoid cells, inflammation

## Abstract

The mechanism of hepatitis B virus (HBV) induced liver inflammation is not fully elucidated. Notch signaling augmented interleukin (IL)-22 secretion in CD4^+^ T cells, and Notch-IL-22 axis fine-tuned inflammatory response. We previously demonstrated a proinflammatory role of IL-22 in HBV infection. Thus, in this study, we analyzed the role of Notch in development of IL-22-producing cells in HBV infection by inhibition of Notch signaling using γ-secretase inhibitor DAPT in both hydrodynamic induced HBV-infected mouse model and in peripheral blood cells isolated from patients with HBV infection. mRNA expressions of Notch1 and Notch2 were significantly increased in livers and CD4^+^ T cells upon HBV infection. Inhibition of Notch signaling *in vivo* leaded to the reduction in NKp46^+^ innate lymphoid cells 22 (ILC22) and lymphoid tissue inducer 4 (LTi4) cells in the liver. This process was accompanied by downregulating the expressions of IL-22 and related proinflammatory cytokines and chemokines in the liver, as well as blocking the recruitment of antigen-non-specific inflammatory cells into the liver and subsequent liver injury, but did not affect HBV antigens production and IL-22 secretion in the serum. Furthermore, IL-22 production in HBV non-specific cultured CD4^+^ T cells, but not HBV-specific CD4^+^ T cells, was reduced in response to *in vitro* inhibition of Notch signaling. In conclusion, Notch siganling appears to be an important mediator of the liver inflammation by modulating hepatic ILC22. The potential proinflammatory effect of Notch-mediated ILC22 may be significant for the development of new therapeutic approaches for treatment of hepatitis B.

## Introduction

Hepatitis B virus (HBV) infection is still a severe public health problem, with ~350 million infections all over the world (Bitton Alaluf and Shlomai, 2016). Current therapies for chronic hepatitis B (CHB) include interferon-α and nucleos(t)ide analogs, which could prevent the progression to end-stage liver diseases. However, cure is rare due to the formation of covalently closed circular DNA within the nucleus of HBV-infected hepatocytes (Zoulim et al., 2016). It is also well accepted that clinical outcome of HBV infection results from the complicated interaction between virus and host immune response (Bertoletti and Ferrari, 2016; Maini and Gehring, 2016), leading to different disease phases (Lok, 2016). Moreover, HBV infection also induces a series of inflammation networks, which manifested as the elevation of specific immune cell populations and molecules (Chang and Lewin, 2007). We previously screened the expression profile of cytokines and chemokines in CHB patients (Lian et al., 2014), and demonstrated the predominate proinflammatory role of interleukin (IL)-22 in HBV infection (Zhang et al., 2011).

IL-22 belongs to IL-10 superfamily of cytokines with potential dual nature of proinflammatory and protective properties (Dudakov et al., 2015). IL-22 was primarily produced by T-helper (Th) 17 and Th22 cells (Perusina Lanfranca et al., 2016), and recent studies have demonstrated a new source of IL-22 in innate lymphoid cells (ILCs) subset, which named as ILC22 (Spits and Di Santo, 2011; Hwang and McKenzie, 2013). Notch signaling is an evolutionally conserved intercellular communication mechanism that controls cell fate decision and differentiation process in a variety of cells (Osborne and Minter, 2007). The interaction between Notch and ligands results in the cleavage of molecules in the transmembrane region by γ-secretase, and followed by intracellular domain translocation into the nucleus (Osborne and Minter, 2007). Increasing evidence indicated that Notch signaling was essential not only in selection of CD4^+^/CD8^+^ lineage choices, but also in controlling of the effector functions of CD4^+^/CD8^+^ T cells (Maekawa et al., 2003, 2008; Radtke et al., 2004; Tsukumo and Yasutomo, 2004). More importantly, Notch signaling up-regulated IL-22 expression in CD4^+^ T cells even in STAT3-deficient manner, and this process was due to Notch-mediated production of aryl hydrocarbon receptor (AhR) stimulator (Alam et al., 2010).

Notch1 was found to be abundantly expressed in CHB patients (Pei et al., 2010). Based on the regulatory mechanism of IL-22 production by Notching signaling (Alam et al., 2010) and the proinflammatory role of IL-22 in HBV infection (Zhang et al., 2011), we hypothesized that Notch signaling performed an important regulatory role for IL-22-producing cells in HBV infection. To test this possibility, we examined the changes in IL-22-producing cells and IL-22 expression as well as their relationship to liver inflammation by inhibition of Notch signaling *in vivo* and *in vitro*.

## Materials and methods

### Mice

Male BALB/c (*H-2*^*d*^) mice 6–8 weeks of age were purchased from Experimental Animal Center of Fourth Military Medical University. The experiments were performed in accordance with the procedures approved by Animal Care and Use Committee of Fourth Military Medical University. Mice were challenged using a hydrodynamic transfection protocol, whereby a total of 10 μg pHBV1.3, with or without 0.1 mg/g of γ-secretase inhibitor DAPT (Selleck Chemicals, Huston, TX, USA), was injected into veins in a volumes of PBS equal to 9% of their body mass (Yang et al., 2002; Cobleigh et al., 2010). Mice were scarified 96 h post-transfection. Livers and serum were harvested for further analysis.

### Virological and biochemical assessments

Semi-quantifications of hepatitis B surface antigen (HBsAg) and hepatitis B e antigen (HBeAg) were performed by electro-chemiluminescence (Architect, Abbott Laboratories, and Abbott Park, IL, USA). Alanine aminotransferase (ALT) level in the serum was measured by Infinity ALT reagent (Thermo Electron, Louisville, CO, USA).

### Flow cytometry

The intrahepatic lymphocytes (IHLs) were purified as described previously (Cobleigh et al., 2010). Purified IHLs were stained with anti-mouse CD3-PerCP Cy5.5, anti-mouse CD4-FITC, anti-mouse CD127-PE Texas Red, anti-mouse NK1.1-PE Cy7, anti-mouse RORγt-PE, and anti-mouse NKp46-APC (all purchased from eBioscince, San Diego, CA, USA). Samples were analyzed with FACS Aria II flow cytometor (BD Biosciences Immunocytometry Systems, San Jose, CA, USA). Acquisitions were performed with CellQuest Pro software (BD Biosciences Immunocytometry Systems), and analyses were performed with FlowJo version 8.7.2 for Windows (Tree Star Inc., Ashland, OR, USA).

### Subjects

A total of 45 patients with HBV infection, including 13 of acute hepatitis B (AHB) and 32 of CHB, were enrolled in the present study. All patients were hospitalized or followed-up between March 2013 and June 2015 in Center for Infectious Diseases of Tangdu Hospital. The standards of diagnoses were made according to the diagnostic standard of Chinese Guideline for Prevention and Treatment of Hepatitis B. Patients who received anti-HBV agents or immunomodulatory medication 1 year before sampling were excluded. No patients were co-infected with human immunodeficiency virus or other hepatitis viruses or concurrently afflicted by immune-compromised diseases or autoimmune disorders. For normal controls (NCs), 20 healthy individuals with matched sex and age were also selected. The clinical characteristics of enrolled subjects were shown in Table 1. The study conformed to the ethical guidelines of the 1975 Declaration of Helsinki. The human study protocol was approved by the Ethics Committee of Tangdu Hospital, and written informed consent was obtained from each participant.

**Table 1:** **Clinical characteristics of enrolled subjects**.

	**AHB**	**CHB**	**NCs**
Cases	13	32	20
Age (year)	29.3 ± 7.5	27.5 ± 7.8	29.4 ± 4.3
Gender (Male/Female)	8/5	22/10	13/7
ALT (U/L)	1784 ± 791	149 ± 28	25 ± 8
HBsAg positive	13	32	0
HBeAg positive	8	32	0
Anti-HBe positive	5	0	0
Anti-HBc IgM positive	13	0	0
HBV DNA (log10IU/mL)	5.32 ± 1.32	6.73 ± 2.05	N.D.

*AHB, acute hepatitis B; CHB, chronic hepatitis B; NCs, normal control; N.D., not determined*.

### Peripheral blood mononuclear cells (PBMCs) isolation and CD4^+^ T cells purification

PBMCs were isolated using Ficoll-Hypaque (Sigma-Aldrich, St Louis, MO, USA) density gradient centrifugation. CD4^+^ T cells were purified using human CD4^+^ T cells isolation kit (Miltenyi Biotec GmbH, Bergisch Gladbach, Germany) under the instruction from manufacturer. The purity of enriched cells for CD4^+^ T cells (>95%) was determined by flow cytometry.

### Cell culture

Purified CD4^+^ T cells were seeded into 24-well plates with concentration of 2 × 10^6^/mL, and were incubated in RPMI 1640 supplemented with 10% of heat-inactivated FBS at 37°C in a 5% CO_2_ environment. CD4^+^ T cells were stimulated by either anti-CD3 antibody (eBioscience, final concentration 1 μg/mL) or HBV core peptides pool (a total of 41 peptides, 15 amino acids of each peptide with 5 amino acids overlapping, final concentration 10 μg/mL), with or without Notch signaling inhibitor DAPT (final concentration 75 μmol/L). Cells and supernatants were harvested 96 h post stimulation.

### Real-time reverse-transcript polymerase chain reaction (RT-PCR)

Total RNA was isolated from cultured CD4^+^ T cells or liver of the mice using RNeasy minikit (Qiagen, Hilden, Germany) according to the instructions from manufacture. RNA was reversely transcribed with random hexamers using PrimeScript RT Master Mix (TaKaRa, Dalian, China). Real-time PCR was performed using SYBR Premix Ex Taq (TaKaRa). Relative gene expression was quantified by ΔΔ*C*_*T*_ method using 7500 System Sequence Detection software (Applied Biosystems, Foster, CA, USA). The sequences of the primers were shown in Table S1.

### Enzyme-linked immunosorbent assay (ELISA)

IL-22 production in the supernatants of cultured CD4^+^ T cells or serum of mice were measured using commercial human/mouse IL-22 ELISA kits (eBioscience) according to the instructions from the manufacture.

### Statistical analysis

Statistical significance was determined by Dunn's multiple comparison test or Student *t*-test using SPSS version 19.0 for Windows (SPSS, Chicago, IL, USA). *P*-values of < 0.05 were considered to indicate a significant difference.

## Results

### Inhibition of notch signaling did not affect HBV antigens production but reduced liver damage *In vivo*

**Figure 1:**
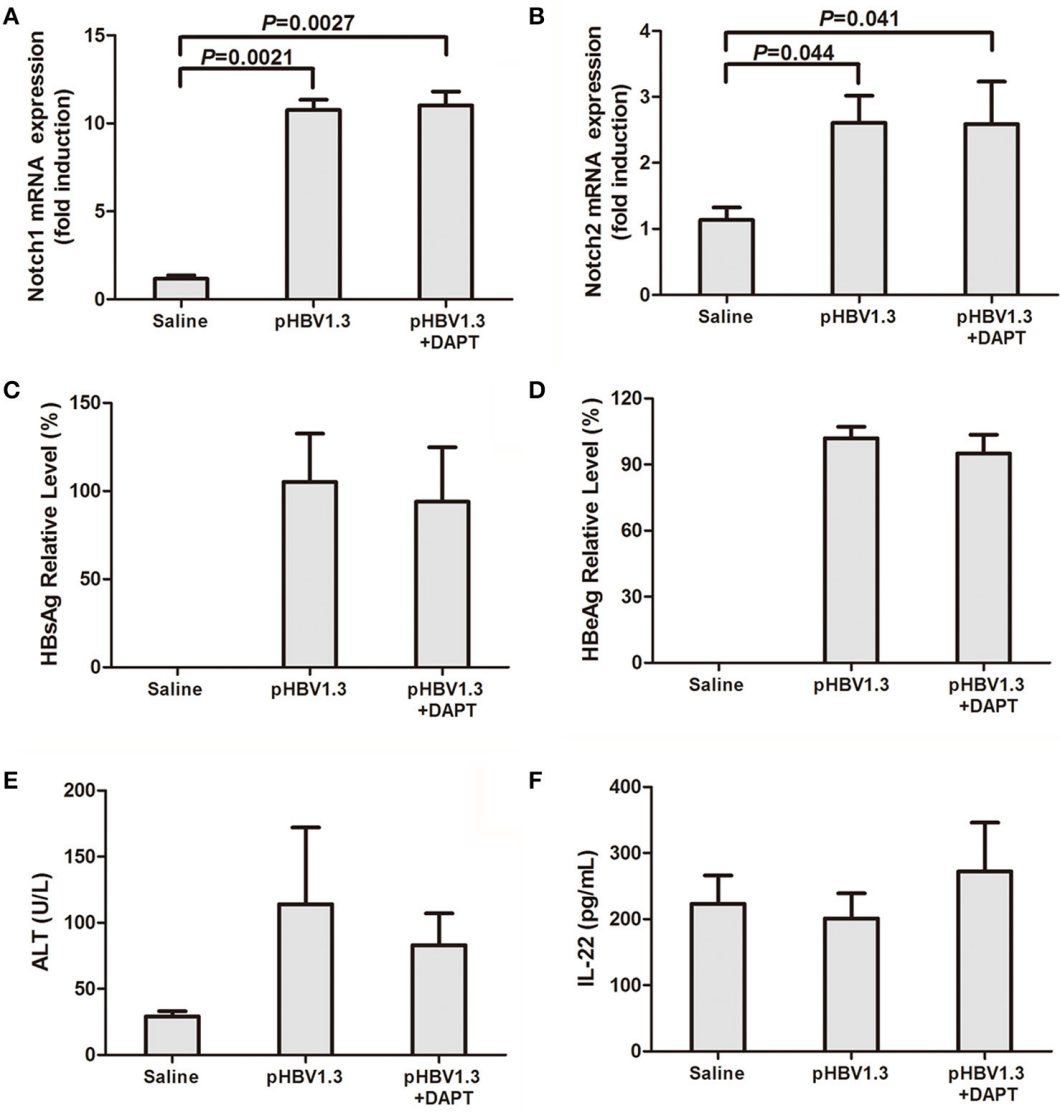
**Inhibition of Notch signaling did not affect HBV antigens production but reduced liver injury**. Mice (4 per group) were hydrodynamically injected with saline, pHBV1.3 plasmid, or pHBV1.3 plus γ-secretase inhibitor DAPT. Mice were scarified 96 h post injection. Livers and serum were harvested for analysis. mRNA expressions corresponding to Notch1 **(A)** and Notch2 **(B)** in the livers were measured by PT-PCR. Results are displayed as fold differences relative to the saline injection group, and normalized to β-actin. HBsAg **(C)** and HBeAg **(D)** in the serum were measured by electro-chemiluminescence. **(E)** Serum ALT levels were measured by Infinity ALT reagent. **(F)** IL-22 concentrations in the serum were tested by ELISA. All values are presented as the average from each group, and error bars represent SE.

We firstly measured Notch mRNA expressions in the liver of the mice. All mice received intravenous injections of saline with or without pHBV1.3 under hydrodynamic conditions, allowing for transfection of hepatocytes *in vivo* (Cobleigh et al., 2010). A group of mice also received hydrodynamic injection combined with pHBV1.3 and Notch signaling inhibitor DAPT. Livers were harvested 96 h post-injection, and Notch mRNA were semi-quantified by RT-PCR. Both Notch1 and Notch2 mRNA expressions were elevated in the liver of mice transfected with pHBV1.3. There were about 10-fold and 2-fold elevations in hepatic Notch1 and Notch2 mRNA in response to HBV plasmid transfection, respectively (Figures 1A,B). In accordance with the elevation of Notch1 and Notch2, HBV plasmid transfection also induced the hepatic mRNA expression of Notch signaling related molecules (Hes1 and Hes5) and Notch ligands [Jagged1, Jagged2, and Delta-like 4 (Dll4)]. DAPT significantly reduced the mRNA expressions of above molecules in the liver, which indicated that DAPT successfully inhibited Notch signaling *in vivo* (Figure S1). However, inhibition of Notch did not downregulate HBV-induced Notch1 and Notch2 expressions in the liver (Figures 1A,B). Furthermore, HBV plasmid transfection induced high levels of HBsAg and HBeAg productions. Blockage of Notch signaling by administration of DAPT did not alter the release of the secreted viral antigens into the serum (Figures 1C,D). Liver damage in mice that received HBV plasmid transfection was moderate as measured by serum ALT level, while DAPT treatment diminished the severity of liver disease with mild elevation of ALT in the serum (Figure 1E). However, there were no remarkable differences among the three groups in the serum concentration of IL-22 (Figure 1F).

**Figure 2:**
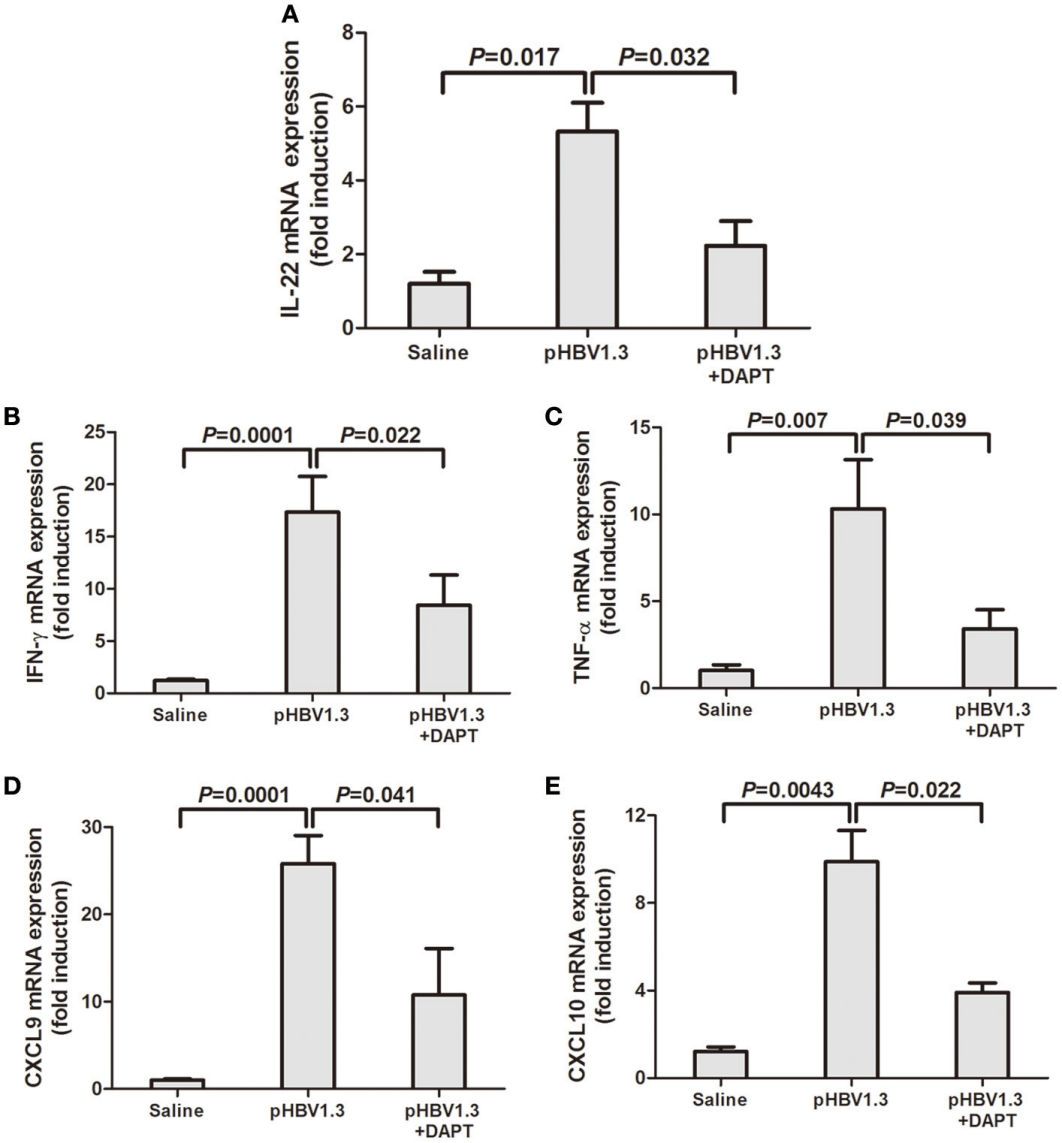
**Inhibition of Notch signaling reduced the HBV-induced elevation of IL-22, and related cytokines and chemokines mRNA expressions in the liver**. mRNA expressions corresponding to IL-22 **(A)**, IFN-γ **(B)**, TNF-α **(C)**, CXCL9 **(D)**, and CXCL10 **(E)** in the livers were measured by PT-PCR. Results are displayed as fold differences relative to the saline injection group, and normalized to β-actin.

We then further measured the IL-22 as well as related cytokines and chemokines in the liver. As shown in Figure 2, IL-22 mRNA expression were significantly elevated in the liver with HBV plasmid hydrodynamic injection, however, inhibition of Notch signaling remarkably reduced the IL-22 mRNA levels (Figure 2A). mRNA expressions of interferon-γ (IFN-γ), tumor necrosis factor-α (TNF-α), CXCL9, and CXCL10 demonstrated similar trends with IL-22 expressions. DAPT treatment suppressed the upregulation of HBV-induced expressions of these cytokines and chemokines (Figures 2B–E).

### Inhibition of Notch signaling reduced the intrahepatic ILC22 subsets and blocked recruitment of inflammatory cells into the liver

**Figure 3:**
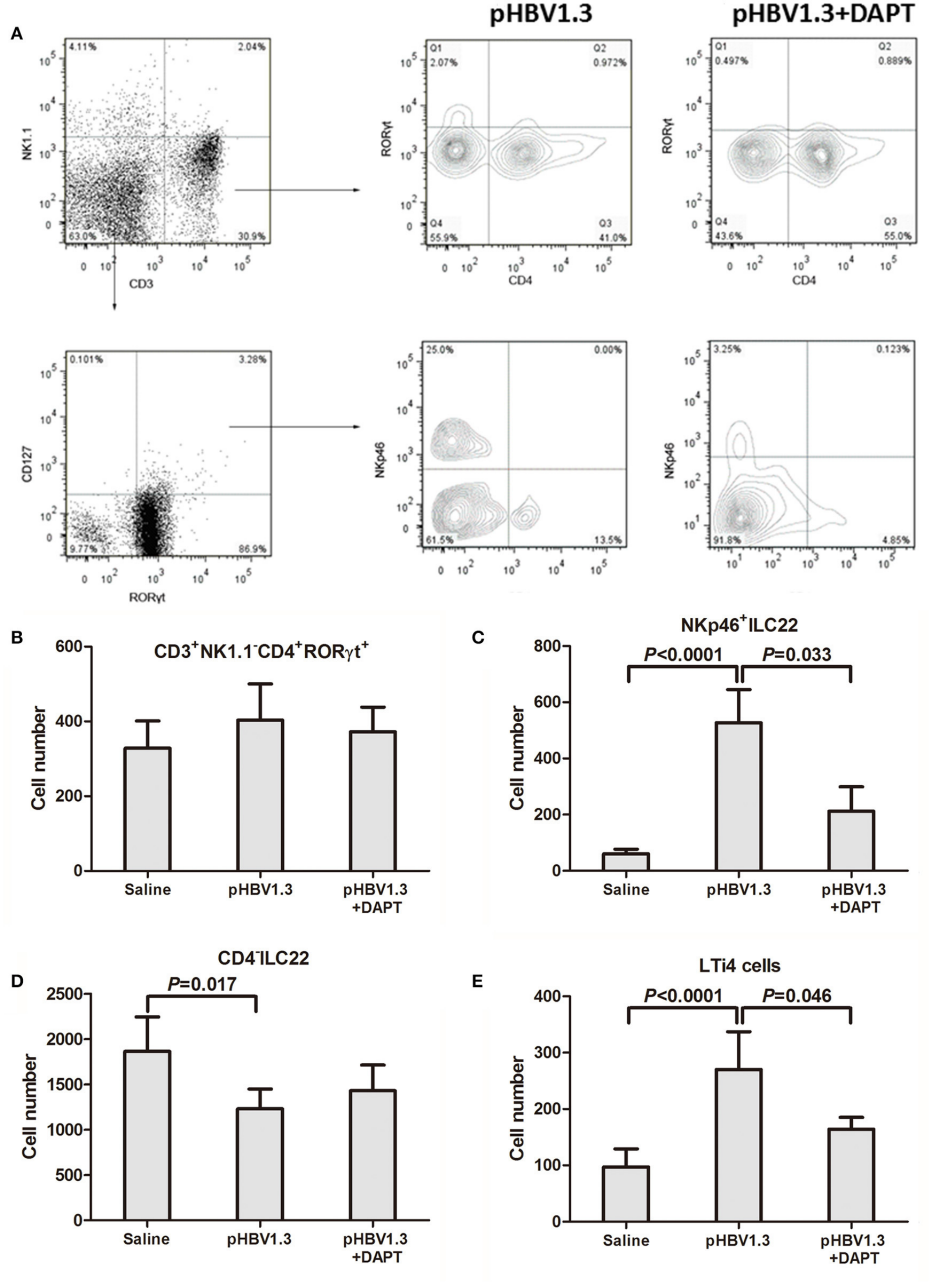
**Inhibition of Notch signaling reduced the intrahepatic NKp46^**+**^ILC22 and LTi4 cells. (A)** Populations in IHLs were discriminated based on expression of CD3, NK1.1, CD127, RORγt, NKp46, and CD4. These included populations of CD3^+^CD4^+^NK1.1^−^RORγt^+^ (mostly Th17 and Th22 cells), CD3^−^NK1.1^−^RORγt^+^CD127^+^NKp46^+^CD4^−^ILC22, and RORγt^+^CD127^−^NKp46^−^ cells (CD4^+^ and CD4^−^). Representative experiments of flow cytometry is shown. Numbers of **(B)** CD3^+^CD4^+^NK1.1^−^RORγt^+^ cells, **(C)** NKp46^+^ ILC22, **(D)** CD4^−^ILC22, and **(E)** LTi4 cells are shown. All values are presented as the average from each group, and error bars represent SE.

We further investigate the changes of IL-22 secreting cells in the liver in response to HBV infection with or without DAPT treatment. We initially focused on the liver resident CD4^+^ T cell population because Th17 and Th22 cells were primary sources of IL-22 (Dudakov et al., 2015). Based on RORγt expression, it is possible to define the CD3^+^NK1.1^−^CD4^+^RORγt^+^ (mostly Th17 and Th22 cells) subset in IHLs (Figure 3A, top). However, we did not observe significant differences in cell numbers of this population among three groups (Figure 3B). Moreover, a specific NKp46^+^ cell subset, which expresses low levels of NK1.1 and named as NKp46^+^ILC22, is known to be an important producer of IL-22 in the intestine (Sanos et al., 2009; Sciume et al., 2012). We therefore analyzed whether those subsets could be found in the liver. Within CD3^−^NK1.1^low/−^RORγt^+^CD127^+^ cells, three subsets can be discerned in IHLs based on CD4 and NKp46 expression: NKp46^+^ILC22 (CD4^−^NKp46^+^), CD4^−^ILC22 [CD4^−^NKp46^−^, CD4^−^ lymphoid tissue inducer (LTi)-like cells], and LTi4 (CD4^+^NKp46^−^) (Sciume et al., 2012; Figure 3A, bottom). Total numbers of NKp46^+^ILC22 and LTi4 increased in the liver of mice with hydrodynamic injection of HBV plasmids (Figures 3C,E). In contrast, CD4^−^ILC22 numbers reduced in response to HBV infection (Figure 3D). DAPT administration reduced the numbers of NKp46^+^ILC22 (Figure 3C) and LTi4 (Figure 3E), only slightly higher than those detected in controls. There was also a consistent trend of elevation in the number of CD4^−^ILC22 with DAPT administration, but this difference failed to achieve significant difference (*P* = 0.092, Figure 3D).

**Figure 4:**
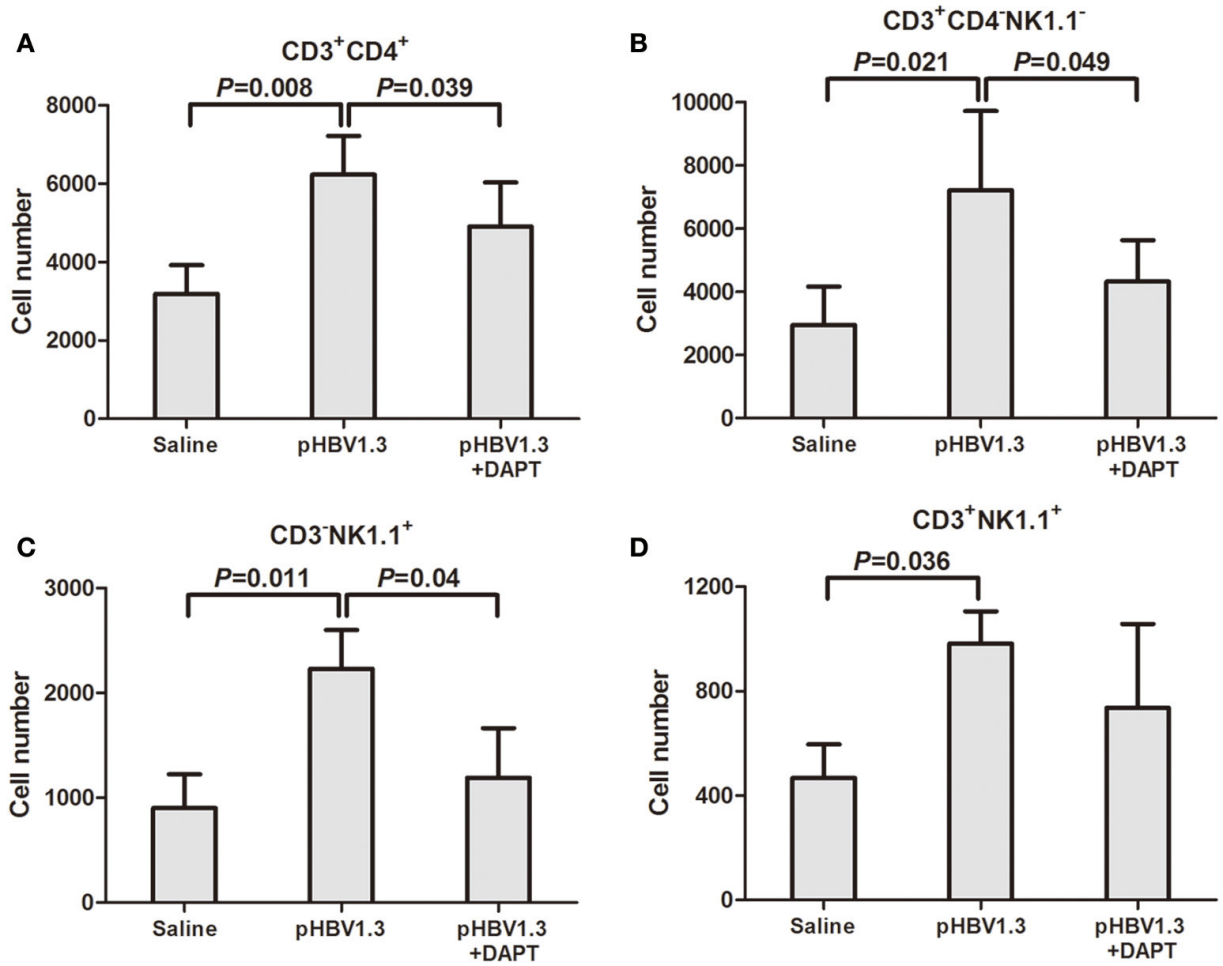
**Inhibition of Notch signaling blocked the recruitment of antigen-non-specific cells into the liver**. IHLs were isolated and analyzed by flow cytometry. **(A)** CD3^+^CD4^+^ cells. **(B)** CD3^+^CD4^−^NK1.1^−^ cells. **(C)** CD3^−^NK1.1^+^ cells. **(D)** CD3^+^NK1.1^+^ cells. All values are presented as the average from each group, and error bars represent SE.

Furthermore, the recruited inflammatory cells into the liver was also determined by flow cytometry. The cell numbers corresponding to Th cells (CD3^+^/CD4^+^, Figure 4A), CTLs (CD3^+^/CD4^−^/NK1.1^−^, Figure 4B), NK cells (CD3^−^/NK1.1^+^, Figure 4C), and NKT cells (CD3^+^/NK1.1^+^, Figure 4D) increased in the liver of mice injected with HBV plasmid. DAPT administration reduced the recruited numbers of Th cells, CTLs, and NK cells with slightly higher numbers than in controls (Figures 4A–C).

### HBV infection induced Notch1 and Notch2 expression in peripheral bloods

**Figure 5:**
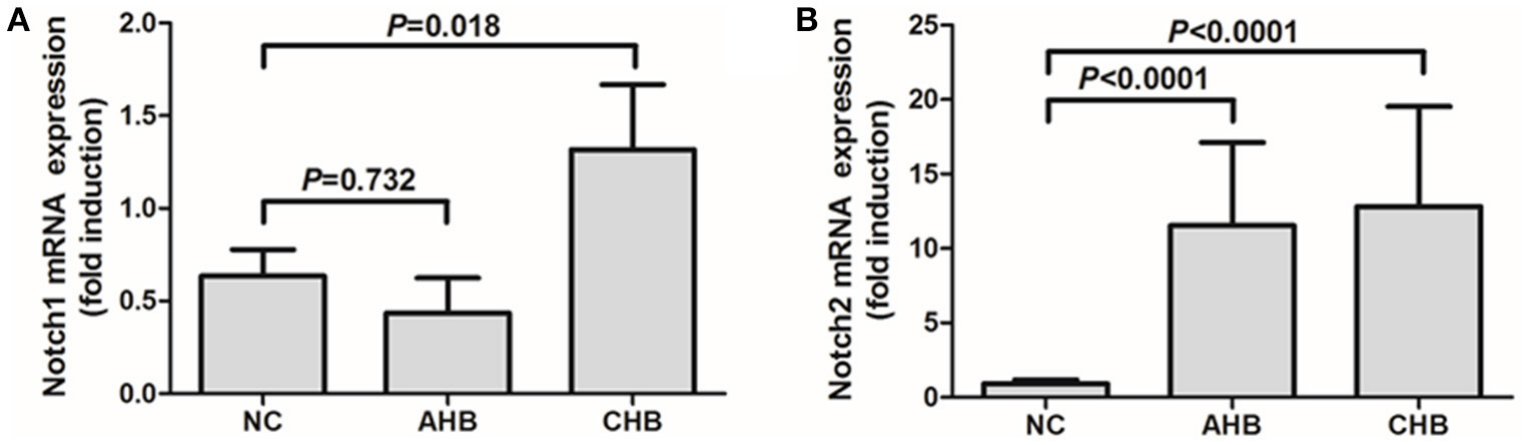
**Expressions of Notch1 and Notch2 were elevated in peripheral CD4^**+**^ T cells isolated from patients with HBV infection**. mRNA expressions corresponding to Notch1 **(A)** and Notch2 **(B)** in CD4^+^ T cells were measured by PT-PCR. Results are displayed as fold differences relative to normal controls, and normalized to 18sRNA. All values are presented as the average from each group, and error bars represent SE.

We then further investigated the Notch-IL-22 axis in human peripheral bloods *in vitro*. Notch1 and Notch2 mRNA expressions in the purified CD4^+^ T cells were investigated by RT-PCR. Notch 1 mRNA expression did not show significant difference between AHB and NCs, however, remarkable increased with approximately 2-fold elevation in CHB in comparison with NCs (Figure 5A). Notch2 mRNA expression were notably elevated with more than 10-fold increase in both AHB and CHB (Figure 5B).

### Inhibition of Notch signaling reduced HBV non-specific IL-22 secretion *In vitro*

**Figure 6:**
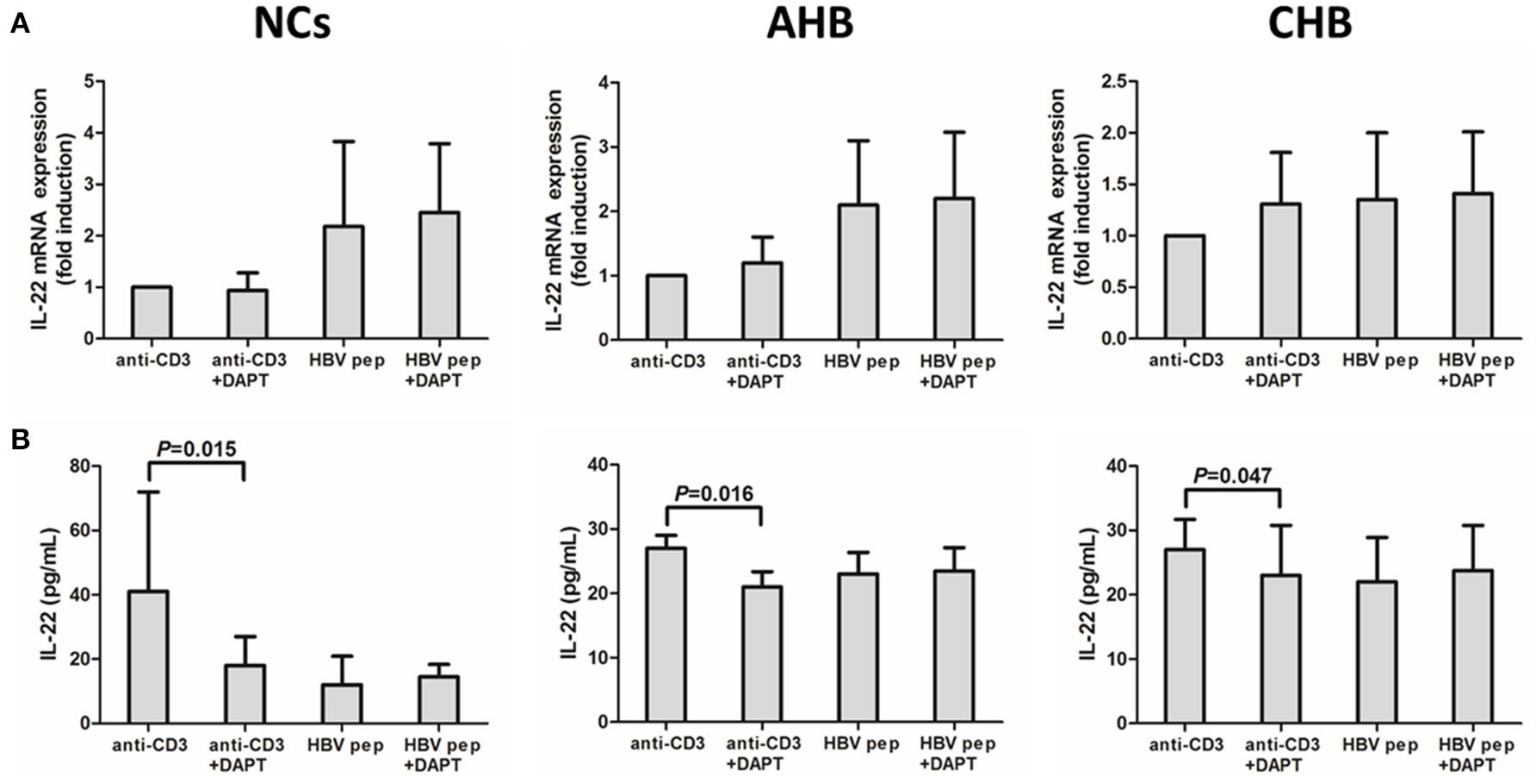
**Inhibition of Notch signaling did not affect IL-22 mRNA expression in isolated CD4^**+**^ T cells, but reduced the HBV non-specific IL-22 production in the supernatants from cultured CD4^**+**^ T cells. (A)** mRNA expressions corresponding to IL-22 in CD4^+^ T cells were measured by PT-CPR. Results are displayed as fold differences relative to anti-CD3 treatment, and normalized to 18sRNA. **(B)** IL-22 production in the supernatants were measured by ELISA. All values are presented as the average from each group, and error bars represent SE.

The purified CD4^+^ T cells were stimulated with either anti-CD3 or HBV core peptides pool, in addition with Notch signaling inhibitor DAPT for 96 h. DAPT significantly reduced the mRNA expressions of both Hes1 and Hes5 in NCs and HBV-infected patients (Figure S2), which indicated that DAPT also successfully inhibited Notch signaling *in vitro*. IL-22 mRNA expression and secretion were then analyzed. DAPT treatment did not affect the IL-22 mRNA expression in CD4^+^ T cells from NCs and HBV-infected patients in both HBV-specific (HBV peptides) and non-specific (anti-CD3) manners (Figure 6A). Furthermore, HBV-specific IL-22 production in the supernatants of cultured cells presented similar levels with or without DAPT treatment (Figure 6B). However, HBV non-specific IL-22 secretion revealed notably reduction in response to DAPT stimulation in both NCs and HBV-infected patients (Figure 6B).

## Discussion

Notch signaling was proven to play a critical role in the pathogenesis of different viral infections. The Notch ligand Dll1 regulated interferon-γ expression of CD4^+^/CD8^+^ T cells, and positively influenced the development of anti-influenza A virus (H1N1) immunity (Ito et al., 2011). Activation of Notch also counteracted with Epstein-Barr virus (EBV) nuclear antigen 2 to inhibit the transcription of key protein in EBV proteins production and the entry of EBV into lytic cycle (Rowe et al., 2014). Repression of Notch receptors mediated the regulation of immune response in CHB patients and the progression to end-stage liver diseases (Trehanpati et al., 2012). In the present study, we found that HBV infection induced Notch1 and Notch2 expression in peripheral blood and liver, consistent with the previous findings of the upregulation of Notch1 expression in CD4^+^ T cells from CHB patients (Pei et al., 2010). Interestingly, recent studies showed that HBV X protein activated Notch signaling via Notch1/Notch4/Dll4 in HBV-associated hepatocellular carcinoma (Gao et al., 2016; Kongkavitoon et al., 2016). However, how Notch signaling regulates the downstream pathway, which contributes to the HBV related inflammation, was still not fully understood.

Notch signaling enhanced IL-22 production by CD4^+^ T cells even in absence of STAT3 by stimulation of AhR in a ConA-mediated acute hepatitis model in RBP-J^−/−^ mice (Alam et al., 2010). Our *in vitro* study also found a reduced expression of IL-22 by HBV non-specific CD4^+^ T cells in response to blockage of Notch signaling, but the mRNA level corresponding to IL-22 did not change significantly. Furthermore, the *in vivo* study showed that inhibition of Notch signaling did not influence HBV antigens production, however, reduced recruitment of inflammatory cells into the liver and subsequent liver injury. Although DAPT treatment did not reduced the ALT levels and IL-22 secretion in the serum, mRNA expressions corresponding to IL-22 as well as related inflammatory cytokines and chemokines in the liver was significantly reduced in response to inhibition of Notch signaling. This was partly because that IL-22-secreting cells are enriched in the tissues but rare in peripheral bloods (Lee et al., 2012). Thus, Notch-IL-22 axis might mainly affect the inflammation in liver-resident, but not in peripheral bloods. This results was also consistent with our previous finding of the proinflammatory function of IL-22 in HBV transgenic mouse model (Zhang et al., 2011), which revealed that Notch signaling also involved in HBV-induced liver injury and inflammation.

We then further investigated the influence of Notch signaling inhibition in the liver resident IL-22-secreting cells. IL-22 was initially thought to be produced in Th17 and Th22 cells (Dudakov et al., 2015). However, we did not find remarkable differences in either numbers or percentages of CD4^+^RORγt^+^ cells (mostly Th17 and Th22 cells) in the liver in response to Notch signaling inhibition. This is partly due to the evidence in RBP-J^−/−^ mice that Notch-mediated IL-22 production was independent of Th17 differentiation (Alam et al., 2010). Moreover, the source of IL-22 was also expanded. ILC22, which mainly localizes in lymphoid tissues of intestinal mucosa, secretes large amounts of IL-22 (Spits and Di Santo, 2011; Sonnenberg et al., 2011a). In the mouse, ILC22 includes two different subsets, NKp46^+^ILC22 and LTi-like cells, with distinct phenotypic markers which reflect functional and developmental heterogeneity (Spits and Di Santo, 2011; Lee et al., 2012). NKp46^+^ILC22 expresses high levels of NK cell marker NKp46, however, low or none of prototypic NK cell marker NK1.1 (Lee et al., 2012). LTi-like cells also include two subsets, CD4^−^LTi (CD4^−^ILC22) and CD4^+^LTi (LTi4) (Sawa et al., 2010; Sonnenberg et al., 2011b). Lee et al. demonstrated that AHR drives the development of gut NKp46^+^ILC22 via induction of Notch, however, LTi cells were partially dependent on Notch signaling (Lee et al., 2012). Herein, we firstly confirmed that all ILC22 subsets (including NKp46^+^ILC22, CD4^−^ILC22, and LTi4 cells) could be detected in the liver of the mouse. HBV infection induced the elevation in numbers of the NKp46^+^ILC22 and LTi4 cells, but reduced CD4^−^ILC22. Inhibition of Notch signaling notably reduced the numbers of NKp46^+^ILC22 and LTi4 cells without downregulation of the HBV antigens expression. Previous study indicated that NKp46^+^ILC22 expressed high level of transcription factor T-bet, which was important for the development and functional regulation of both NK cells and NKp46^+^ILC22 (Sciume et al., 2012), however, limited expression of T-bet was found in CD4^−^ILC22 and LTi4 cells. There were few reports in the regulation and function of tissue-resident CD4^−^IL-22 cells. van de Pavert et al. (2014) showed that cell-autonomous retinoic acid signaling controlled mouse fetal ILC3, including ILC4neg cells (CD3^−^CD127^−^α4β7^+^ID2^+^c-Kit^+^CD11C^−^CD4^−^) and LTi4 cells. However, the function and regulation of liver-resident CD4^−^ILC22 need to be further elucidated. These results indicated that Notch signaling was important in regulation of liver NKp46^+^ILC22 and LTi4 cells in HBV infection, but not sufficient to directly influence virus itself *in vivo*.

Due to the potential dual nature of proinflammatory and protective roles of IL-22, we examined the predominate function of ILC22 in the intrahepatic inflammatory response in HBV infection. Although the study suggested Notch-mediated IL-22 protected RBP-J^−/−^ mice from ConA-induced hepatitis (Alam et al., 2010), more recent study on experimental autoimmune uveoretinitis revealed a key pathological role of RBP-J/Notch-induced IL-22 production in late phase of the disease (Bhuyan et al., 2014). Furthermore, Song et al. demonstrated NKp46^+^ILC3 subsets, which mainly produced IL-17 and IL-22, promoted inflammation through granulocyte-macrophage colony stimulating factor-induced accumulation of inflammatory monocytes (Song et al., 2015). In this study, we also showed that inhibition of Notch signaling reduced the recruitment of some kinds of inflammatory cells into the liver and ameliorated liver inflammation. This process was along with the suppression of NKp46^+^ILC22 and LTi4 cells. Thus, ILC22 may therefore contribute to the HBV induced liver disease pathogenesis by promoting the migration of inflammatory cells into the liver. However, IL-22 levels in the serum was not remarkably decreased. This may partly due to the limited numbers of hepatic ILC22, which was not enough for secreting sufficient IL-22 to the blood stream and could only worked in the liver resident surroundings.

In summary, we found that Notch signaling is dispensable for HBV antigens secretion, but important in modulation of hepatic NKp46^+^ILC22 and LTi4 cells, which may potentiate the intrahepatic recruitment of antigen non-specific cells and increase liver injury. A similar role of Notch-IL-22 axis may also be played in the pathogenesis in patients with HBV infection. The potential proinflammatory effect of Notch-mediated ILC22 may be significant for the development of new therapeutic approaches for treatment of hepatitis B.

## Author contributions

Design and supervise the study: YZ and JL. Perform the experiments: XW, JW, CQH, and XY. Enroll the patients: CQH, LW, CXH, XB, and JL. Interpret and analyze the data: LW, CXH, XB, JL, and YZ. Prepare the manuscript: XW, JL, and YZ.

## Funding

This work was supported by the grants from National Natural Science Foundation of China (81671555 and 31200650), National Science and Technology Major Project of China (2012ZX10002-001-006 and 2016ZX10002010-011), Wang Bao-En Research Foundation for Liver Fibrosis in China Foundation for Hepatitis Prevention and Control (2014016), and a grant from Tangdu Hospital (supported YZ).

### Conflict of interest statement

The authors declare that the research was conducted in the absence of any commercial or financial relationships that could be construed as a potential conflict of interest.
