# Supplementary material for: Notch Signaling Contributes to Liver Inflammation by Regulation of Interleukin-22-Producing Cells in Hepatitis B Virus Infection
Source: Front Cell Infect Microbiol. 2016 Oct 17;6:132. doi: 10.3389/fcimb.2016.00132 (PMC5065963; doi:10.3389/fcimb.2016.00132)
Supplement: Supplementary file 1 [file DataSheet1.PDF]

Supplementary Material

Notch signaling contributes to liver inflammation by regulation of interleukin-22-producing cells in hepatitis B virus infection

Xin Wei [1#], Jiu-Ping Wang [2#], Chun-Qiu Hao [1#], Xiao-Fei Yang [1], Lin-Xu Wang [1], Chang-Xing Huang [1], Xue-Fan Bai [1], Jian-Qi Lian [1]*, Ye Zhang [1]*

[#] Xin Wei, Jiu-Ping Wang, and Chun-Qiu Hao contributed equally to this work.

* Correspondence **Ye Zhang:** zhangyefmmu@hotmail.com

Jian-Qi Lian: lianjq@fmmu.edu.cn

Table S1. Primer sequences for RT-PCR

Primers	Sequences
human Notch1 sense	5’-TCA GCG GGA TCC ACT GTG AG-3’
human Notch1 anti-sense	5’-ACA CAG GCA GGT GAA AGA CGA GTT G-3’
human Notch2 sense	5’-GGC ATT AAT CGC TAC AGT TGT GTC T-3’
human Notch2 anti-sense	5’-GGA GGC ACA CTC ATC AAT GTC A-3’
human Hes1 sense	5’-GGA CAT TCT GGA AAT GAC AGT GAA-3’
human Hes1 anti-sense	5’-AGC GCA GCC GTC ATC TG-3’
human Hes5 sense	5’-AGG GCG AGC CTG CTC CTC TC-3’
human Hes5 anti-sense	5’-CGG AGA AGG GCG CGT CCA AG-3’
human IL-22 sense	5’-GCA GGC TTG ACA AGT CCA ACT-3’
human IL-22 anti-sense	5’-GCC TCC TTA GCC AGC ATG AA-3’
human 18sRNA sense	5’-CGC CGC TAG AGG TGA AAT TC-3’
human 18sRNA anti-sense	5’-TTG GCA AAT GCT TTC GCT C-3’
mouse Notch1 sense	5’-TGC CAG GAC CGT GAC AAC TC-3’
mouse Notch1 anti-sense	5’-CAC AGG CAC ATT CGT AGC CAT C-3’
mouse Notch2 sense	5’-ACG CCA CCT GCC TGG ATA AG-3’
mouse Notch2 anti-sense	5’-CAC CTG CCC GTT GTT CAC AC-3’
mouse Hes1 sense	5’-AAA GAC GGC CTC TGA GCA C-3’
mouse Hes1 anti-sense	5’-GGT GCT TCA CAG TCA TTT CCA-3’
mouse Hes5 sense	5’-CTG GAG ATG GCC GTC AGC TA-3’
mouse Hes5 anti-sense	5’-GTA GTC CTG GTG CAG GCT CTT G-3’
mouse Jagged1 sense	5’-AAA GGC TTC ACC GGC ACC TAC-3’

mouse Jagged1 anti-sense	5’-CTC CCA GCC GTC ACT ACA GAT ACA-3’
mouse Jagged2 sense	5’-CCT GAV AGC AGC CTG ATC CA-3’
mouse Jagged2 anti-sense	5’-ACT GAA CAC GCT GCA CAG CAC-3’
mouse Dll1 sense	5’-GAC ACC AAG TAC CAG TCG GTG TAT G-3’
mouse Dll1 anti-sense	5’-AAC CTG GTT CTC AGC AGC AGT C-3’
mouse Dll4 sense	5’-CTG TGA GCT GGG ACT CAG CAA G-3’
mouse Dll4 anti-sense	5’-ATG CTC ACA GTG CTG GCC ATA G-3’
mouse IL-22 sense	5’-AAC TAA CCC CCT TTC CCT GC-3’
mouse IL-22 anti-sense	5’-AAC GCA GGG GTT CAT TTG GA-3’
mouse IFN-γ sense	5’-CTC TGA GAC AAT GAA CGC TAC-3’
mouse IFN-γ anti-sense	5’-TTC TTC CAC ATC TAT GCC ACT-3’
mouse TNF-α sense	5’-TGC CTC AGC CTC TTC TCA TT-3’
mouse TNF-α anti-sense	5’-TGG AAG ACT CCT CCC AGG TA-3’
mouse CXCL9 sense	5’-GAT CAA ACC TGC CTA GAT CC-3’
mouse CXCL9 anti-sense	5’-GGC TGT GTA GAA CAC AGA GT-3’
mouse CXCL10 sense	5’-ACC ATG AAC CCA AGT GCT GCC GTC-3’
mouse CXCL10 anti-sense	5’-GCT TCA CTC CAG TTA AGG AGC CCT-3’
mouse β-actin sense	5’-CAT CCG TAA AGA CCT CTA TGC CAA C-3’
mouse β-actin anti-sense	5’-ATG GAG CCA CCG ATC CAC A-3’

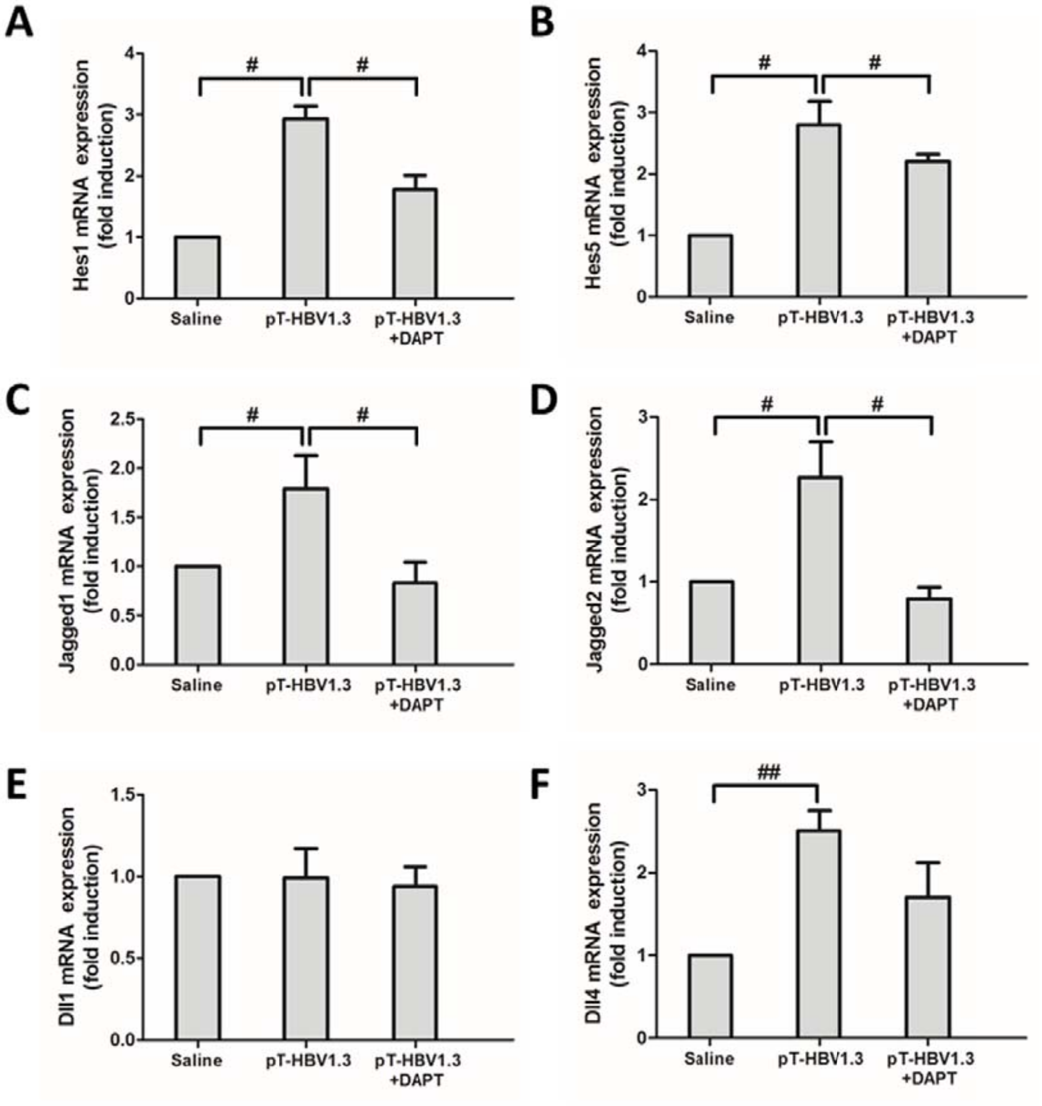

Figure S1. Inhibition of Notch signaling reduced the expressions of Notch signaling related molecules and Notch ligands in the liver of mice. mRNA levels corresponding to (A) Hes1, (B) Hes5, (C) Jagged1, (D) Jagged2, (E) Dll1, (F) Dll4 were measured by RT-PCR. Results are displayed as fold differences relative to the saline injection group, and normalized to β-actin. All values are presented as the average from each group, and error bars represent SE.

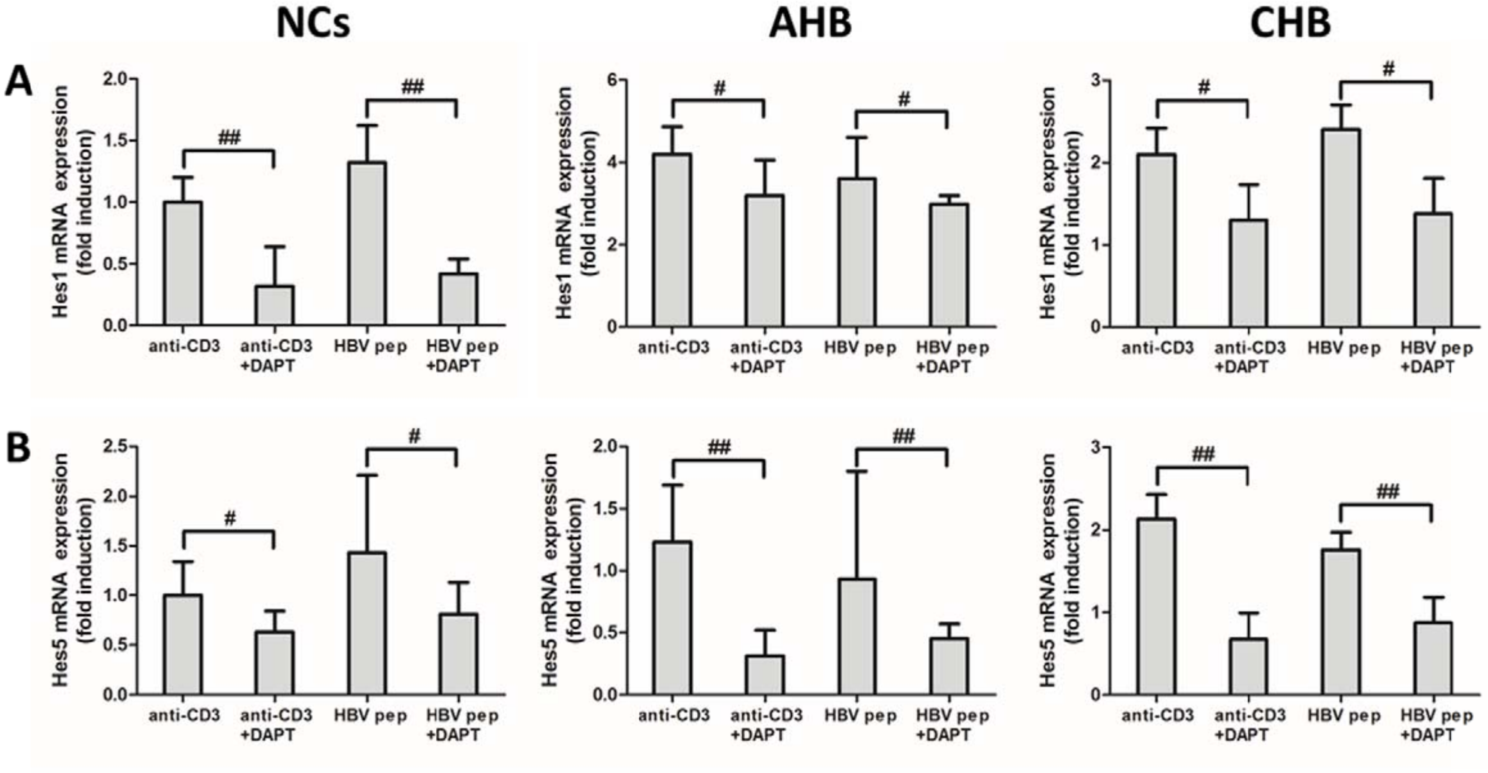

Figure S2. Inhibition of Notch signaling reduced Hes1 and Hes5 mRNA expression in cultured CD4$^+$ T cells isolated from normal controls (NCs), acute hepatitis B (AHB), and chronic hepatitis B (CHB). mRNA levels corresponding to (A) Hes1 and (B) Hes5 were measured by RT-PCR. Results are displayed as fold differences relative to anti-CD3 treatment, and normalized to 18sRNA. All values are presented as the average from each group, and error bars represent SE.
